# Using Triplet Ordering Preferences for Estimating Causal Effects in the Analysis of Gene Expression Data

**DOI:** 10.1371/journal.pone.0170514

**Published:** 2017-01-31

**Authors:** Alexander K. Hartmann, Grégory Nuel

**Affiliations:** 1 Institut für Physik, Universität Oldenburg, 26111 Oldenburg, Germany; 2 LPMA, CNRS 7599, Université Pierre et Marie Curie, Paris, France; Pennsylvania State University, UNITED STATES

## Abstract

Triplet ordering preferences are used to perform Monte Carlo sampling of the posterior causal orderings originating from the analysis of gene-expression experiments involving observation as well as, usually few, interventions, like knock-outs. The performance of this sampling approach is compared to a previously used sampling via pairwise ordering preference as well as to the sampling of the full posterior distribution. For a fair comparison, the latter approach is restricted to twice the numerical effort of the triplet-based approach. This is done for artificially generated causal, i.e., directed acyclic graphs (DAGs) and for actual experimental data taken from the ROSETTA challenge. The sampling using the triplets ordering turns out to be superior to both other approaches.

## Introduction

For the last 10 years, high-throughput omics data have raised many methodological challenges in system biology. Among these challenges, gene-regulation networks have received a great deal of attention. In this context, Gaussian models like the Graphical lasso [1] or approaches based on mutual information [2] are very popular for inferring gene regulation networks. In case time-resolved data is available, e.g., dynamic Bayesian networks [3] or ordinary differential equations [4] can be applied. Another popular approach, following the work of Pearl [5], focuses on causal Gaussian Bayesian networks and performs intervention calculus [6] proving itself to be able to retrieve bounds on causal effects and thus to partially determine causal relationships using only observational data [7]. In this paper we focus on estimating causal Bayesian networks in the presence of arbitrary mixtures of (non-time resolved) observational and interventional data [8, 9], i.e., wild-types and knock-out/down experiments with possibly multiple interventions within each experiment.

As explained in [8] estimating the underlying DAG (Directed Acyclic Graph) structure of a causal Bayesian network is equivalent to finding of the so-called *causal ordering* between the genes of interest. In general, this causal ordering is unknown and belongs to a very large ordering space (*p*! possible orderings for *p* genes) which cannot be explored exhaustively. The solution suggested by [8] consists in sampling causal orderings in the posterior distribution using Markov chain Monte-Carlo (MCMC) simulations.

At each MCMC step, a new causal ordering is sampled according to a proposal distribution (ex: Mallows distribution) and the maximum likelihood of the model must be computed given the new ordering before to accept/reject the sampled ordering. Thanks to the closed formulas developed in [8], this likelihood maximization can be done exactly and efficiently but requires a computational effort which still grows with the sixth power of the number *p* of interacting objects (ex: genes). Thus, each single Monte Carlo step is computationally rather expensive.

Mathematically a proper MCMC is guaranteed to converge to the correct sampling, but only on diverging time scales. Given that for practical applications one only has a finite amount of computational resources available, only small networks can be treated in this way. For this reason, an approximation based solely on pairwise probabilities of ordering preference has recently been introduced [10]. This resulted in a considerable increase of efficiency, but led in many cases to less reliable parameter estimates.

In this work, we extend this approximation to triplet-wise probabilities. We show that this results in a strongly increased accuracy with respect to the pairwise approach. Also we show that, when allocating a comparable amount of the numerical resources for the two algorithms, the triplet approach outperforms the sampling based on the full maximum likelihoods. Thus, the triplet algorithm is well balanced: it is sophisticated enough to allow for a rather accurate sampling, while it is computationally cheap enough to be applicable in practice.

The reminder of this work is organized as follows: In Section “Model” we introduce the model we use to analyze causal relationships and state all algorithms we have applied. In Section “Results” we introduce the quantities we have measured to compare the different approaches, and we present the corresponding results. We conclude in Section “Summary and Discussion” with a summary and discussion.

## Model and Algorithms

### Model

We consider directed graphs *G* = (*V*, *E*) with *p* nodes *i* ∈ *V*. Pairs of nodes *i*, *j* are connected by directed edges (*i*, *j*) ∈ *E* and carry a weight *w*_*i*,*j*_. The matrix **W** = (*w*_*i*, *j*_) contains all weights. A nonzero weight indicates a causal relationship. We assume that the graph is acyclic, i.e., a directed acyclic graph (DAG). Without loss of generality, we can assume that the nodes are ordered according the causal relationships, i.e., *w*_*i*,*j*_ > 0 ⇒ *i* < *j*. This means within the following random process only nodes *i* can have causal effects on nodes *j* if *i* < *j*:

On each node *j* = 1, …, *p* a Gaussian random variable *X*_*j*_ is placed given by

$$X_{j}=m_{j}+\sum_{i<j}w_{i,j}X_{i}+\epsilon_{j}\;\text{with}\;\epsilon_{j}∼N\left(0,\sigma_{j}^{2}\right)\;.$$ (1)

Thus, the term *ϵ*_*j*_ is responsible for the fluctuations of the variables, e.g., for fluctuations of gene expression and $\sigma_{j}^{2}$ describes the level of fluctuations. In particular, the parameters **m** = (*m*_1_, …, *m_p_*) and *σ* = (*σ*_1_, …, *σ_p_*) represent the mean values and the standard deviations if all interactions were absent. In the following an *experiment* corresponds to one realization of the random process Eq (1).

Within the model it is, furthermore, possible to perform *interventions* on the nodes, i.e., within selected but arbitrary realizations of the process they are fixed to given values instead of generated according to Eq (1). In the DAG these values are used as inputs to the descendants when generating a realization of the process, i.e., performing an experiment numerically [11].

### Estimating Model Parameters

Given are *N* experimental data points $x^{k}=\left(x_{1}^{k},\ldots ,x_{p}^{k}\right)$ (1 ≤ *k* ≤ *N*) assumed to be generated according to Eq (1). The set of nodes subject to interventions on experiment *k* is denoted by *J*_*k*_, respectively (*J*_*k*_ = ∅ means no intervention for the *k*’th experiment). We denote by *K*_*j*_ = {*k*|*j* ∉ *J*_*k*_} the experiments where there was no intervention on node *j* and by *N*_*j*_ = |*K*_*j*_| the number of times nodes *j* were not target of an intervention. The log-likelihood of the joint experimental outcome given the parameters has been derived in detail in Ref. [8]. Here we only state the final result for brevity:

$$\ell \left(m,\sigma ,W\right)=-\frac{\log(2\pi )}{2}\sum_{j}N_{j}-\sum_{j}N_{j}\log\left(\sigma_{i}\right)-\frac{1}{2}\sum_{j}\frac{1}{\sigma_{j}^{2}}\sum_{k\in K_{j}}{\left(x_{j}^{k}-x^{k}We_{j}^{T}-m_{j}\right)}^{2}\;,$$ (2)

where $e_{j}^{T}$ denotes the transpose of the unit vector which has a value 1 in position *j* and zero entries everywhere else. Note in order to write this equation in the standard form of the multinomial distribution, one uses the covariance matrix **Σ** = **L**^*T*^ diag(*σ***^2^**)**L**, where **L** = (**I** − **W**)^−1^ and **I** = diag(**1**) [8]. The above stated form is more convenient, because it is already diagonal. We omit the dependence of *ℓ* on the data here for brevity of notation. For the given *N* measurements, the parameters $\hat{m},\hat{\sigma },\hat{W}$ leading to the maximum likelihood estimator (MLE)

$$\ell_{\max}=\ell \left(\hat{m},\hat{\sigma },\hat{W}\right)=\max_{m,\sigma ,W}\ell \left(m,\sigma ,W\right)$$ (3)

can be obtained [8] in a straightforward way by the following procedure: First one obtains for each experiment *k* = 1, …, *N* the measurements normalized with respect to the experiments where there was no intervention on nodes *j*, for each node *j*:

$$y^{k,j}=x^{k}-\frac{1}{N_{j}}\sum_{k^{\prime }\in K_{j}}x^{k^{\prime }}.$$ (4)

Next one solves the linear system of size *p*(*p* − 1)/2

$$\sum_{i^{\prime }|i^{\prime }<j}{\hat{w}}_{i^{\prime },j}\sum_{k\in K_{j}}y_{i}^{k,j}y_{i^{\prime }}^{k,j}=\sum_{k\in K_{j}}y_{i}^{k,j}y_{j}^{k,j}\;\text{for}\;i<j,\;1\leq i,j,\leq N$$ (5)

to obtain estimates ${\hat{w}}_{i,j}$ of the weights for the MLE. Solving a linear system with *O*(*p*^2^) variables takes *O*(*p*^6^) steps. From this solution one obtains, still just following [8], estimates of the mean values

$${\hat{m}}_{j}={\frac{1}{N}}_{j}\sum_{k\in K_{j}}\left(x_{j}^{k}-x^{k}\hat{W}e_{j}^{T}\right)$$ (6)

and of the variances

$${\hat{\sigma }}_{j}={\frac{1}{N}}_{j}\sum_{k\in K_{j}}{\left(y_{j}^{k,j}-y^{k,j}\hat{W}e_{j}^{T}\right)}^{2}\;.$$ (7)

### Estimating the Posterior Distribution

So far, we have assumed that the causal ordering of the model is given by **o**_0_ = (1, 2, …, *p*). In experimental situations, if the data was actually generated according the DAG model, the ordering is most of the time unknown, i.e., all estimates will depend on the ordering: *ℓ*_max_ = *ℓ*_max_(**o**). for the general case, if the data was not generated according to a DAG model, the modeling must involve many orderings. Thus, in experiments and subsequent model estimation, one is actually interested in either the ordering which maximizes the MLE, or, alternatively, in obtaining the posterior distribution involving all (or the dominant) orderings weighted by the corresponding ordering-dependent MLEs.

Both can be obtained in principle by iterating over all *p*! possible causal orderings **o**, i.e., permutations of the natural numbers 1, …, *p*. Each time one has to reorder the measurement data according this ordering, and obtaining the MLE Eq (3) via solving Eqs (4), (5), (6) and (7). Clearly, if *p* is too large, this enumeration is not possible any more.

One alternative approach is to use a *Markov-chain Monte Carlo* (MCMC) simulation, where orderings **o**(*t*) according the likelihood exp(*ℓ*_max_(**o**)) are sampled, *t* denotes the number of steps. A convenient approach to achieve this is the *Metropolis algorithm*. Here, within each step, a *trial order* **o**′ is generated. For the present study, we use local changes, i.e., an exchange of the order of two nodes with respect to the current ordering **o**(*t*). The trial ordering is *accepted*, i.e., **o**(*t* + 1) = **o**′ with the probability

$$p_{\text{acc}}=\min\left\{1,\exp\left[\ell_{\max}\left(o^{\prime }\right)-\ell_{\max}\left(o\left(t\right)\right)\right]\right\}\;.$$ (8)

Otherwise, the trial ordering is not accepted and the current ordering kept for the next time step, i.e., **o**(*t* + 1) = **o**(*t*). Note that for all simulations we performed (see below for details), the empirical acceptance rate of these locally generated trial orderings was below 0.5. The value of 0.5 is considered by rule of thumb as a good choice, balancing a desired high rate of changing with a desired high acceptance rate. Therefore it would not make sense to consider trial orderings which differ from the current ordering by more than two exchanged positions, since this would increase the fluctuations and therefore decrease the acceptance rate even more.

This type of sampling guarantees, in principle, if the Markov chain is long enough, that the orderings are sampled according the desired posterior distribution. Note that for the computation of the change *ℓ*_max_(**o**′) − *ℓ*_max_(**o**(*t*)) of the log-likelihood one has to recalculate the log-likelihood for the trial ordering **o**′ from scratch. Thus, each MCMC Metropolis step takes *O*(*p*^6^) running time.

By starting with a random ordering **o**(0), performing a “long enough” MCMC sampling and by discarding the “initial” part (allowing for *equilibration*), a sample set *S* of orderings is obtained, which can be used to calculate averaged estimated parameters, see Section “Calculation of Averaged Estimates”.

### Calculation of Averaged Estimates

The aim is to study expectation values in ensembles defined by probabilities or likelihoods *P*(**o**). Here we are interested in the true likelihoods $P\left(o\right)∼e^{\ell_{\text{max}}\left(o\right)}$. Thus, for any measured quantity *A*(**o**), where the estimate depends on the assumed ordering **o**, the expectation value is given by

$$\langle A\rangle \equiv \sum_{o}A\left(o\right)P\left(o\right).$$ (9)

Note that the measured quantities of interest are usually estimates which are obtained from the maximum-likelihood calculation, e.g., the estimates of the weights obtained from Eq (5) or estimates of the variances Eq (7), or any other derived values.

If only a finite set *S* of samples is given, averages can be obtained, approximating the expectation values:

$$\hat{A}\equiv \frac{\sum_{o\in S}A\left(o\right)P\left(o\right)}{\sum_{o\in S}P\left(o\right)}$$ (10)

These estimates are most accurate if the process use to generate the sample set follows the desired sampling $P\left(o\right)∼e^{\ell_{\text{max}}\left(o\right)}$ as close as possible. Thus, the sample set *S* could be generated by a MCMC sampling according to the true probabilities $e^{\ell_{\text{max}}}$, as outlined in the previous section. In this way automatically orderings with high contributions to Eq (10) are preferentially generated. Note that since *S* is actually a mathematical *set*, there will be no multiple occurrences of orderings in *S*. If one allowed for multiple occurrence, then one would have to take simple arithmetic averages instead of weighted ones as in Eq (10).

Anyway, here we work with sampling sets. The reason is that, alternatively, these sets can be obtained by sampling according to different probabilities, which only aim at approximating the true probabilities but which are computationally much cheaper to calculate. If the size of the set is suitably restricted, we used always |*S*| = 100, the computationally expensive *O*(*p*^6^) full likelihood calculations have to be performed only for a small number of (here) 100 samples.

The approximate probabilities we have used are introduced in the following section.

### Pair and Triplet Probabilities

Instead of sampling the full posterior distribution, in [10] it was proposed to perform an MCMC sampling from a different distribution, the Babington-Smith (BS) ordering distribution [12, 13]. It is based on pair preferences *π*_*i*,*j*_ (1 ≤ *i* ≠ *j* ≤ *p*) with *π*_*i*,*j*_ ∈ [0, 1] and *π*_*i*,*j*_ + *π*_*j*,*i*_ = 1. The meaning is that within the desired ordering distribution in any random ordering element *i* appears before *j* with this probability *π*_*i*,*j*_. The pair preferences can be estimated from the experimental data with interventions by considering all possible two-node graphs *G*_*i*,*j*_ ≡ ({*i*, *j*}, {(*i*, *j*)}) with the nodes *i* and *j* and with exactly one directed edge (*i*, *j*). As above, for brevity of notation, we omit the dependence of the pair preferences and any derived quantities on the data here. Only the data values for the two nodes are considered. Note that in case of multiple interventions, we observed in tests, which are not contributing to the results shown here, that the overall performance of the sampling according pair preferences is somehow better if data points with interventions on other nodes than *i*, *j* are not included in the dataset for the pair *i*, *j*, respectively. For each of the *p*(*p* − 1) directed two-node graphs the log-likelihood $\ell_{\text{max}}^{\left(2\right)}\left(i,j\right)$ is obtained. The pair preferences are then given by

$$\pi_{i,j}=\frac{\exp(\ell_{\text{max}}^{\left(2\right)}\left(i,j\right))}{(\exp(\ell_{\text{max}}^{\left(2\right)}\left(i,j\right)+\exp\left(\ell_{\text{max}}^{\left(2\right)}\left(j,i\right)\right)}.$$ (11)

From the pair preferences, the BS probability of a full ordering **o** is obtained by

$$P\left(o|\pi \right)∼\prod_{i<j}\pi_{o_{i},o_{j}}\;$$ (12)

with a suitable normalization. The normalization is not needed here, since, first, we only compare the (relative) values of Eq (12) for different orderings. The corresponding log-likelihoods are denoted as

$$\ell^{\text{pair}}\equiv \ell^{\text{pair}}\left(o\right)=\log\prod_{i<j}\pi_{o_{i},o_{j}}\;.$$ (13)

Second, we performed MCMC sampling of orderings using the Metropolis algorithm according Eq (12) where also only relative likelihoods are needed. This was done in an equivalent way as above, only the true MLE is replaced by Eq (13). Thus, starting again from a random ordering **o**(0), we generated trial orderings **o**′ by exchanging the *i*’th and the *j*’the entry in the current ordering. The new orderings are accepted with the corresponding Metropolis probability. Note that one does not have to recalculate the BS probability from scratch, since the change in probability is easier to obtain. The Metropolis acceptance probability is given by

$$p_{\text{acc}}^{\text{pair}}=\min\left\{1,\frac{\pi_{o_{j},o_{i}}}{\pi_{o_{i},o_{j}}}\prod_{k|i<k<j}\frac{\pi_{o_{j},o_{k}}\pi_{o_{k},o_{i}}}{\pi_{o_{i},o_{k}}\pi_{o_{k},o_{j}}}\right\}\;.$$ (14)

This takes only *O*(*p*) steps compared to the *O*(*p*^2^) steps which are needed for the calculation of the full probability. In particular it is much faster than computing the full likelihood which takes *O*(*p*^6^) steps.

Naturally, when sampling according to Eq (12) the observed set of orderings will be different but somehow similar to when sampling according the true likelihood. The reason is that orderings with a high full likelihood induce in principle large pairwise probabilities for those pairs which are compatible with such an order. Nevertheless, the pairwise approximation cannot completely cover collective effects which involve the ordering of more than two nodes. Thus, the final estimates, like the weights, for the posterior distribution are obtained by keeping the *n*_incl_ samples with the highest Babington-Smith probabilities Eq (12) in the sample set *S*. For these orderings now the *true MLE* Eq (3) is evaluated and used. This means, Eq (10) is applied for any kind of estimation or averaging, i.e. the Babington-Smith weights are now used in this final averaging step.

In [10] it was found that this sampling approach is in some case similar accurate as a full MCMC sampling as described in Sec. Estimating the Posterior Distribution, but there were notable differences. In particular when the number *p* of nodes is growing, the orderings exhibiting the largest pair-based probabilities turned out to be more and more different from the orderings exhibiting large full likelihoods. This showed up in particular when calculating estimates of network parameters. Therefore it was proposed to maybe consider triplets instead of pairs.

Thus, it is the purpose of the present work, to study this higher level approximation of the true posterior distribution. Similar to the above defined pair probabilities, we introduce triplet probabilities *ρ*_*i*,*j*,*k*_ ∈ [0, 1] such that *ρ*_*i*,*j*,*k*_ + *ρ*_*i*,*k*,*j*_ + *ρ*_*j*,*i*,*k*_ + *ρ*_*j*,*k*,*i*_ + *ρ*_*k*,*i*,*j*_ + *ρ*_*k*,*j*,*i*_ = 1. These probabilities can be estimated from the experimental data in a similar way as above, by considering all possible sub graphs ({*i*, *j*, *k*}, {(*i*, *j*), (*i*, *k*), (*j*, *k*)}) with three nodes and corresponding edges. For these sub graphs the corresponding MLE are obtained and suitably normalized, equivalent to Eq (11) to yield the triplet probabilities *ρ*_*i*,*j*,*k*_. They can be used to generalize the Babington-Smith probabilities of orderings to

$$P\left(o|\rho \right)∼\prod_{i<j<k}\rho_{o_{i},o_{j},o_{k}}\;.$$ (15)

Again, the normalization is not needed here. The corresponding log-likelihood is denoted as

$$\ell^{\text{tripl}}=\log\prod_{i<j<k}\rho_{o_{i},o_{j},o_{k}}\;.$$ (16)

We perform an MCMC sampling of orderings according these probabilities using the Metropolis algorithm and trial ordering generated via swapping of pairs of elements. Like for the case of sampling according the pair-based probabilities, these swapping pairs are chosen unbiased, i.e., each pair is chosen with the same probability. Therefore, to guarantee a sampling according to Eq (15), for the calculation of the acceptance probabilities only the change in probability of Eq (15) has to be considered, which now takes *O*(*p*^2^) steps for such a swap.

Again, for all evaluation and estimations, the *n*_incl_ = 100 highest-probability samples with respect to the triplet probability are kept. For these samples the true likelihood is obtained and used for all averaging processes according to Eq (10).

### Data Sources

The new approach will be tested and compared to previous approaches using data from biological applications as well for data generated my numerical simulations for DAGs of different sizes.

For the latter one, we consider random DAGs with *p* nodes. For the edge weights, each edge (*i*, *j*) with *i* < *j* receives independently a zero weight with probability 1 − *q*, i.e., these edges are absent. With probability *q* each edge gets assigned an edge weight which is drawn uniformly from the range [−1, −0.4] ∪ [0.4, 1]. Thus, these edge can be distinguished very well from the absent edges with weight 0. Below, we use *q* = 1, i.e. complete graphs, as well as diluted graphs with *q* = *c*/(*p* − 1), i.e., these graphs have on average *c* neighbors. We used *c* = 6. Finally, for each DAG instance, for each node *j* mean values *m*_*j*_ = 1/2 are used and the variance values *σ*_*j*_ are drawn randomly uniformly in the interval [0.01, 0.1]. We also performed some tests with other values and verified that our general conclusions do not all depend on how the means and the standard deviations are chosen. Below we will also show one set of results for very strong randomness *σ*_*j*_ ∈ [0.1, 1]. Thus, for the majority of the nodes the fluctuations are stronger than the mean for this case. All simulations are performed for 1000 DAG instances generated independently in this way.

Next, for each DAG instance, a certain number of *N* measurements is performed, where the measurement vectors $x^{k}$ (*k* = 1, …, *N*) are generated according to Eq (1). Typically, for a DAG of *p* nodes, we generated *N* = 10*p* measurement vectors, other cases are stated when it applies. We used a variable number of interventions to investigate how the different sampling approaches respond to that variation. Note that the scheme exhibited in Section “Estimating Model Parameters” allows for multiple intervention. Nevertheless, since we are interested in comparing different sampling approaches here, we present for simplicity just single interventions which are systematically done the first *r* (*r* ≤ *N*) experiments of each set of experiments. We applied a systematic manner, such that for all nodes at least ⌊*r*/*p*⌋ interventions are performed while for *r* − ⌊*r*/*p*⌋ nodes one intervention more, i.e., ⌈*r*/*p*⌉ interventions are performed. This sums up to *r* interventions. For each intervention on node *j*, we set *X_i_* = 0, respectively, corresponding to a knock-out.

The advantage of using artificially generated data is that the actual model used to generate the data is available. Therefore all estimated and averaged values, obtained using a sampling via the true likelihoods as well as using a sampling based on pair and triplet probabilities, can be compared to the actual model parameters. This allows for a good comparison of the different sampling approaches. In particular for a varying number of network sizes, even large ones, and for varying number of interventions.

**Fig 1:**
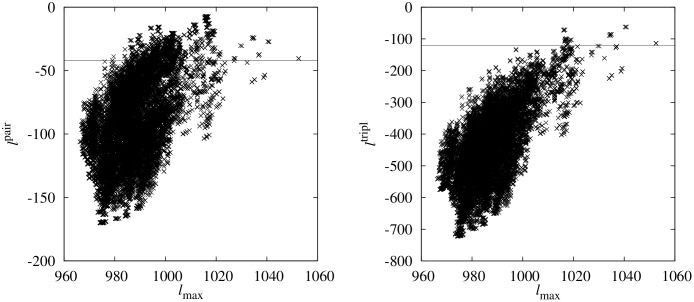
Log-likelihood comparisons. Left: Scatter plot of the true log-likelihood Eq (2) versus the pairwise log likelihood Eq (13) for data generated for a DAG of size *p* = 8. All 8! orderings were enumerated and the pairs of true likelihood and pair probability plotted. The horizontal line indicates the pairwise BS log-likelihood for that ordering which exhibits the maximum true likelihood. Right: The same but for true log-likelihood (x-axis) versus triplet log-likelihood Eq (16). Note the different scales of the pairwise and triple-wise log-likelihoods are only due to the missing normalization of likelihoods.

On the other hand, the DAG models might not represent all subtleties of biological applications. Thus, to allow for a different viewing angle on the different approaches, we also applied data obtained from biological measurements. Here, we used the Rosetta Compendium data set [14] which contains gene expression data on yeast. It contains data from experiments on mutants with interventions (knock-out or know-down) for single as well as multiple interventions. Also a large amount of data from wild-type experiments (no interventions) is contained. The database can be accessed freely at the location: http://arep.med.harvard.edu/ExpressDB. We used in particular a sub network taken from [15] consisting of *p* = 17 genes (STT2, TEC1, NDJ1, KSS1, YLR343W, YLR334C, MFA1, STE6, KAR4, FUS1, PRM1, AGA1, AGA2, TOM6, FIG1, FUS3, YEL059W) and data for *N* = 300 experiments. For this set of genes, no knowledge about any possibly underlying network structure or network parameters is assumed while performing the numerical tests here. Only the actual experimental outcomes taken from the database are used. Thus, the estimated parameters generated using the true likelihood from the set of reference values here to perform a comparison of the different approaches. For this purpose, to allow for an exact enumeration, avoiding sampling errors for the reference values, we selected [10] a sub network consisting of *p* = 8 genes, namely STT2, TEC1, NDJ1, KSS1, YLR343W, YLR334C, MFA1, STE6. Note that these genes form a coherent sub network in the network estimated in [15]: with respect to the full 17 nodes network, only one single interaction involving a node of the sub network STE6 (to FUS1) is missing for this selected subset. For these 8 genes, four of the experiments contained single node interventions, namely knock-downs on nodes KSS1, SST2, and twice on TEC1.

## Results

To evaluate and compare the power of the Babington-Smith pair and triple approaches, we applied them to various data obtained from DAG ensembles of different graph sizes as well as to data obtained from biological applications.

First, as shown in Section “Direct Comparison”, we applied the calculation of the Babington-Smith pair and triple likelihoods to a single graph, where we enumerated all orderings and compared the result to the full likelihoods. In Section “Application to Rosetta Compendium”, the results of the application of the MCMC sampling to the Rosetta data set are shown. Next, in Section “Evaluation for Random DAGs”, MCMC sampling for all three types of likelihoods, respectively, were applied to data obtained for different random DAGs of size *p* = 20 and *p* = 50 nodes. Finally, in Section “Greedy Approach”, the results of estimating the most likely orderings via greedy algorithms based on pair and triplet probabilities for random DAGs are shown.

### Direct Comparison

First, we evaluated the likelihood computation for a single randomly picked realization of a complete (*q* = 1) DAG with *p* = 8 nodes. We performed *N* = 100 experiments, among those *r* = 4 with single-node interventions. For this sample, we enumerated all *p*! = 40,320 orderings, and for each ordering we evaluated the true likelihood Eq (2) together with the pairwise Babington-Smith log-likelihood Eq (13) and the triplet-wise BS log-likelihood Eq (16).

In the left part of Fig 1, for each ordering the pairwise Babington-Smith likelihood is shown as a function of the full likelihood. This means a scatter plot of *p*! orderings of points (*ℓ*_max_(**o**), *ℓ*^pair^(**o**)) is shown. The ordering **o**^max^ leading to the maximum full likelihood appears to the right of the scatter plot, with *ℓ*_max_(**o**^max^) ≈ 1053. This ordering will dominate any average according to Eq (10). Obviously, this ordering does not exhibit the maximum pairwise BS likelihood, which is *ℓ*^pair^ ≈ −7, obtained by an ordering which true log-likelihood is about *ℓ*_max_ ≈ 1016. The horizontal line in the plot indicates the pairwise BS log-likelihood *ℓ*^pair^ of the ordering **o**^max^. A considerable number of all orderings, actually more than 2700, are located above this line. Thus, they exhibit a value of *ℓ*^pair^ which is higher than for the ordering **o**^max^. Therefore, when performing an MC sampling according the pairwise likelihoods Eq (13) plus evaluating the true likelihoods for averaging, one must generate a very large sample if the true maximum-likelihood ordering is to be included.

The corresponding result considering triple-wise BS likelihoods *ℓ*^tripl^ is shown in the right part of Fig 1 in the same way. Here, the sequence exhibiting the maximum value of *ℓ*^tripl^ ≈ −62 has a true log-likelihood of *ℓ*_max_ ≈ 1040, which is much closer to the sequence **o**^max^ which has (still) *ℓ*_max_(**o**^max^) ≈ 1053. Only about 100 sequences exhibit a triple-wise BS likelihood larger than for **o**^max^ (indicated again by the horizontal line). This means an MC sampling using the triplet-wise BS likelihoods allows for much more accurate estimation of model parameters with respect to the true likelihoods. This can be seen also in the next section, where an actual biological application is considered.

### Application to Rosetta Compendium

For the experimental data points of the Rosetta Compendium for *p* = 8 nodes and *N* = 300 experiments with four interventions (see Section “Data Sources” for details), we obtained the averages of the estimated interaction parameters according to Eq (10). One can either estimate the direct causal effects, i.e., the entries *w_ij_* of the weight matrices **W**. Here, we concentrated on the matrix

$$L=1+W+W^{2}+\ldots +W^{p-1}={\left(I-W\right)}^{-1},$$ (17)

which carries the total (direct and indirect) causal effects [10] mediated through chains of causal effects (note that **W**^*p*^ = 0 because of the DAG structure). Thus, for all cases, we estimated the 8 × 8 = 64 entries of the matrix **L**.

The sampling was performed in four different ways:

1. All *p*! = 40320 orderings were enumerated and the true expectation value for all 64 matrix entries was obtained via Eq (9).
2. To estimate the influence of a finite sample size, a subset *S* of 1000 orderings with the highest true likelihoods $e^{\ell_{\text{max}}\left(\sigma \right)}$ was taken. For this subset the averages of estimates of the 64 matrix entries were obtained via Eq (10).
3. An MCMC sampling according the pair BS probabilities Eq (12) was performed.1000 independent MCMC chains were performed, each starting with an independently chosen random ordering. The length of each MCMC chain consisted of 100 pair-exchange trial steps according to Eq (14). From these orderings, the set *S* of the 1000 orderings exhibiting the highest pair BS probabilities was taken and the average estimates of the matrix entries were obtained via Eq (10).
4. An MCMC sampling according the triplet BS probabilities Eq (12) is performed, in an equivalent way as for the pair BS probabilities. All parameters were the same and the analysis was performed in the same way. Thus, everything was the same, except that the pair BS probabilities were replaced by the more demanding triplet BS probabilities.

**Fig 2:**
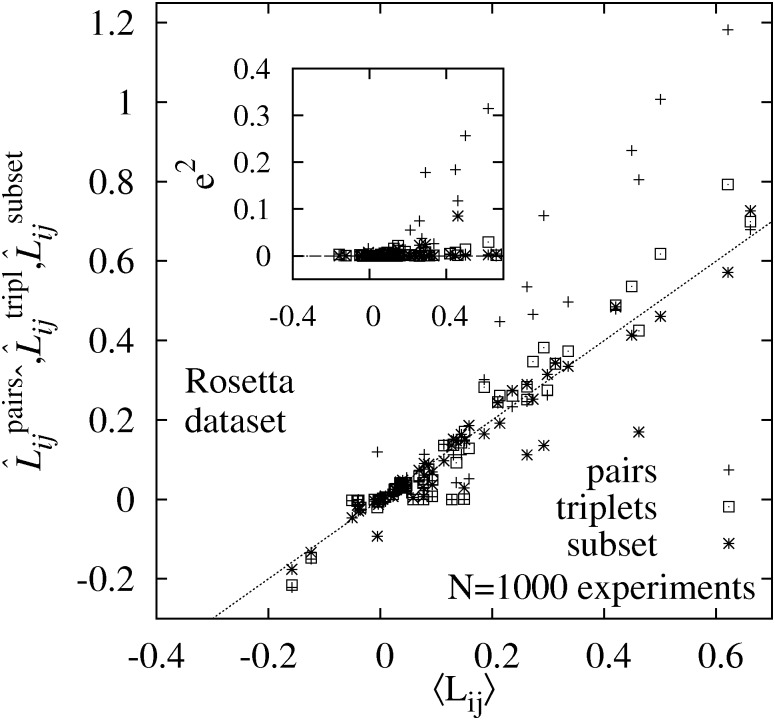
Comparison on the Rosetta Dataset. Comparison of the estimations of the 64 entries of the total causal effects matrix **L** using the exact expectation values 〈*L*_*ij*_〉 (from a complete enumeration) and estimates ${\hat{L}}_{ij}$ obtained from the three approaches: pairwise sampling, triplet-wise sampling, and a subset of the exact sample. For each matrix entry, the average value obtained via one of the three approaches is shown, respectively, as a function of the exact expectation value. The data is taken from the module Rosetta data set (8 genes). The inset shows the mean-squared error *e*^2^ between averaged entry and exact expectation value, as a function again of the exact expectation values.

In Fig 2 the averages obtained from the approaches 2–4 are compared to the exact expectation values obtained from the first approach. For a perfect estimation of the averages, all data points would lie on the diagonal. Clearly deviations are visible, which is to be expected since the averages are only approximations of the expectation values. The main result is that the deviations are much stronger for the sampling using the pair probabilities. On the other hand, for the triplet probabilities, the scatter of the data points is comparable to the scatter of the exact sampling of restricted size. This shows that the sampling of a finite size set of ordering samples is already close to perfect when using the triplet probabilities.

In the inset of Fig 2 we also show the mean-squared errors $e^{2}={\left({\hat{A}}^{a}-\langle A\rangle \right)}^{2}$, where *A* are the different matrix entries *L*_*ij*_ and ‘a’ denotes the algorithm (a = pairs, triplets, subset). The above findings are supported by MSE values, which are comparable for triplets probability and subset (exact probability) sampling, but much larger for the pair probability sampling.

### Evaluation for Random DAGs

Next, we show results for numerically generated data for an ensemble of DAGs. This has the advantage that due to the average the influence of fluctuations is negligible when comparing the efficiencies of the different sampling approaches. Furthermore, we were able to perform the simulations for different DAG sizes, here we studied DAGs with *p* = 20 and with *p* = 50 nodes. Also, we could vary the number *r* of interventions over a wide range to get a grip on how these influences the performance of the different algorithms. Finally, we could compare the estimated parameters with the original values used to generate the data. Thus, to measure the efficiency, we consider all edge weights *w*_*i*,*j*_, where *w*_*i*,*j*_ might be zero because it does not match the causal ordering, or because the causal interaction is just absent (in the case of edge probability *q* < 1). This is done in the following way: From each sampling, we obtain averaged estimated edge weights ${\hat{w}}_{i,j}$ (*i*, *j* = 1, …, *p*) according to Eq (10). Now, we count the “bad” estimates of the edge weights as follows:

$$\delta_{\text{bad}}\left(i,j\right)=\left\{\begin{array}{cc} \Theta (|{\hat{w}}_{i,j}|-w_{0}) & \text{if}\;w_{i,j}=0\\ \Theta \left(\left|\frac{w_{i,j}-{\hat{w}}_{i,j}}{{\hat{w}}_{i,j}}\right|-w_{1}\right) & \text{if}\;w_{i,j}\neq 0 \end{array}\right.\;.$$ (18)

Θ(*x*) denotes the threshold function which is Θ(*x*) = 0 for *x* ≤ 0 and Θ(*x*) = 1 for *x* > 0. Thus, for a weight which is zero in the original DAG used to generate the data, the averaged estimate is counted as bad if its absolute value exceeds a threshold value *w*_0_. For an edge with nonzero weight of the original DAG the average estimate is counted as bad, if the relative deviation of the average estimated weight and the original weight exceeds threshold value *w*_1_. We used *w*_0_ = 0.1 and *w*_1_ = 0.5. In general, details of the results might depend on the actual values of *w*_0_ and *w*_1_, but we verified that the principal trends, with respect to which sampling approach performs better, remain the same. To exclude the influence of the actual threshold values, we also performed a *Receiver Operator Characteristics* (ROC) analysis, see below. We iterated over all edges, i.e. measured

$$n_{\text{bad}}=\frac{1}{p(p-1)}\sum_{i\neq j}\delta_{\text{bad}}\left(i,j\right)\;.$$ (19)

The results we show are an average over all 1000 random DAGs.

The measurement data was obtained for *N* = 10*p* experiments, i.e., *N* = 200 experiments for *p* = 20 nodes and *N* = 500 experiments for *p* = 50. We performed interventions for a varying number 0 ≤ *r* ≤ *N* of experiments as explained in Section “Data Sources”. The different sets *S* of sampled orderings, for which the averages ${\hat{w}}_{i,j}$ were calculated using Eq (10), were obtained via four different sampling approaches, respectively:

pairs An MCMC sampling according the pair BS probabilities Eq (12) is performed. 100 independent MCMC chains were performed, each starting with an independently chosen random ordering. The length of each MCMC chain consisted of 10100 pair-exchange trial steps according to Eq (14). During the last 100 steps of each MCMC chain, configurations were stored, i.e., the initial 10000 steps are for equilibration. From these 10000 stored orderings, the set *S* of the 100 orderings exhibiting the highest pair BS probabilities was taken and the average entries, now using the true maximum likelihoods of these configurations, were obtained via Eq (10).
triplets An MCMC sampling according the triplet BS probabilities Eq (12) is performed, in an equivalent way as for the pair BS probabilities. All parameters were the same and the analysis was performed in the same way. Thus, everything was the same, except that the pair BS probabilities were replaced by the more demanding triplet BS probabilities.
full In a similar way a MCMC sampling with the full maximum likelihoods was performed. Here only 10 independent runs starting with random orderings were done. Note hat in the limit of infinite long simulation time, each of such an MCMC chain should yield the true expectation values Eq (9). Nevertheless, for a fair comparison, the length of the MCMC chains was chosen such that the full simulation CPU time was slightly above two times the running time of the MCMC simulation using the triplet BS probabilities. Since each MCMC step involves a full *O*(*p*^6^) calculation of the maximum likelihoods, this means per MCMC chains only 50 steps could be performed.
exact The set *S* consisted only of the original ordering of nodes which was used generate the data. Thus, only one single *O*(*p*^6^) maximum likelihood computation has to be performed. This usually yielded the best estimates of the parameters. Clearly, in true experiments, this ordering is not available.

**Fig 3:**
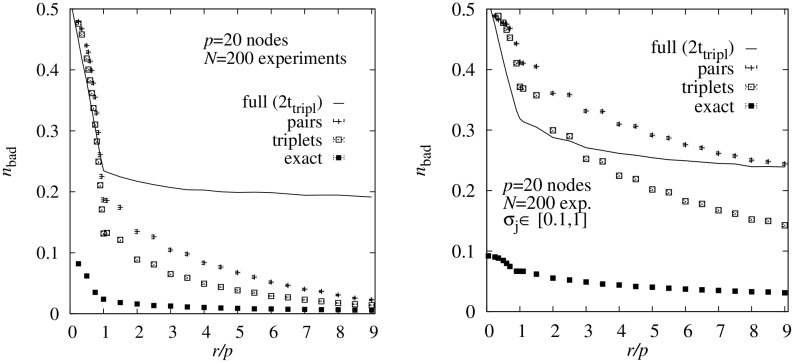
Topological errors. Average fraction *n*_bad_ of incorrectly estimated edge weights as a function of the number of interventions *r* per node. The data was generated for 1000 randomly generated DAGs of size *p* = 20 nodes. The results are obtained using four different sampling approaches using the true maximum likelihoods (**full**), the pair BS probabilities (**pair**), the triplet BS probabilities (**triplet**) and using just the exact ordering of nodes of the DAGs. The running time for the sampling using the true maximum likelihoods was restricted to two times the CPU time of the triplet sampling. The left plot shows the result for standard deviation *σ*_*j*_ drawn from the interval [0.01, 0.1] (as for almost all results presented here) while the right plot is for strong fluctuations with *σ*_*j*_ ∈ [0.1, 1].

In Fig 3 (left) the resulting average values for the fraction *n*_bad_ of incorrectly estimated edge weights is shown as a function of the relative number *r*/*p* of single-node interventions. One can observe that with increasing number of interventions, the quality of the averaged weight estimate increases. This is especially true for the range *r* < *p* where the number of interventions is smaller than the number of nodes in the DAG. For *r* > *p* the quality of the averaged estimates increases only slightly.

Also one can observe that the full sampling, due to the limited number of MCMC steps performed, is the worst approach, except for a very small number of interventions, where the estimates are bad anyway. Furthermore, the quality of the estimates is much better when using the triplet probabilities as compared to the pair probabilities. Still, one cannot reach the quality of the estimate which we obtained when using the single true ordering. Thus, the result from the true ordering constitutes a lower limit for what is possible using sampling.

To give an impression of the influence of the fluctuations, we also show in Fig 3 (right) the corresponding results for the case of large fluctuations, with standard deviations *σ*_*j*_ drawn in the interval [0.1, 1]. This is very strong compared to the mean values *m*_*j*_ = 1/2. The overall picture remains the same, only that the pair approximation becomes very bad now and that it is beneficial to use more interventions. In terms of efficiency versus effort, the triplet approach comes out best.

As mentioned already, the details of the results for *n*_bad_ depend on the choice of the threshold values *w*_0_ and *w*_1_. For this reason we also show how here the results change when varying *w*_0_ or *w*_1_ for a fixed value *r*/*p* = 5, see Table 1. The relative order of the approaches is the same for all combinations of the threshold parameters: Using the exact ordering (which is usually not available) is the best. The triplet-based approach is better than the pair-based. Using the full likelihood with the same numerical effort is worst. Note that, naturally, the fraction *n*_bad_ decreases when increasing the thresholds, because the criterion is easier to fullfill. Only for the case of changing *w*_0_ the result for the “exact” approach does not change, because the true ordering is used which means that all zero edges are correct by default, for any threshold *w*_0_.

**Table 1:** Values for the fraction *n*_bad_ of incorrectly estimated edge weights for *p* = 20, *N* = 200, *r*/*p* = 5 for various values of the threshold *w*_0_ and *w*_1_. The results for the standard threshold value *w*_0_ = 0.1 and *w*_1_ = 0.5 are included in the third row.

threshold		*n*_bad_			
*w*_0_	*w*_1_	full	pairs	triplets	exact
0.02	0.5	0.221	0.073	0.041	0.009
0.05	0.5	0.208	0.070	0.039	0.009
0.1	0.5	0.199	0.067	0.038	0.009
0.2	0.5	0.192	0.066	0.037	0.009
0.1	0.1	0.310	0.187	0.156	0.122
0.1	0.2	0.265	0.124	0.090	0.053
0.1	0.8	0.164	0.048	0.024	0.002

To obtain results which are independent of the actual choice of the thresholds, we furthermore determined the ROC for whether a weight is considered non-zero or not. For this purpose we used a simple thresholding, i.e., a weight for edge *i*, *j* is considered non-zero if its estimate exceeds a threshold ${\hat{w}}_{i,j}\geq w_{2}$. Thus, for a large threshold value, only few weights will be considered as nonzero, while for a small value of *w*_2_ many weights will be considered as non-zero. Since we know the weights used to generate the data, we know those edges which are correctly identified as being non-zero, i.e., the number of *true positives* *N*_pos_, as well as the number of incorrectly as being non-zero identified edges, the *false positives* *N*_false_. For the corresponding normalized rates *n*_pos_ = *N*_pos_/*p*^2^ and *n*_false_ = *N*_false_/*p*^2^, the function *n*_pos_(*n*_false_) can be obtained by varying *w*_2_. This is the actual ROC curve. The steeper it grows for small values of *n*_false_, i.e., the more true positives are found at the cost of accepting false negative estimates, the better is the determination of the non-zero edge weights. Thus, the *area* *A*_ROC_ under the ROC (AUROC) is a measure for the quality of the estimate. Since the AUROC is a number obtained via the variation of the threshold *w*_2_ it has the advantage of being parameter-free. Due to the normalization, the AUROC is bounded by one, which is the optimum case of finding all true positive non-zero weights without false-positive ones.

**Fig 4:**
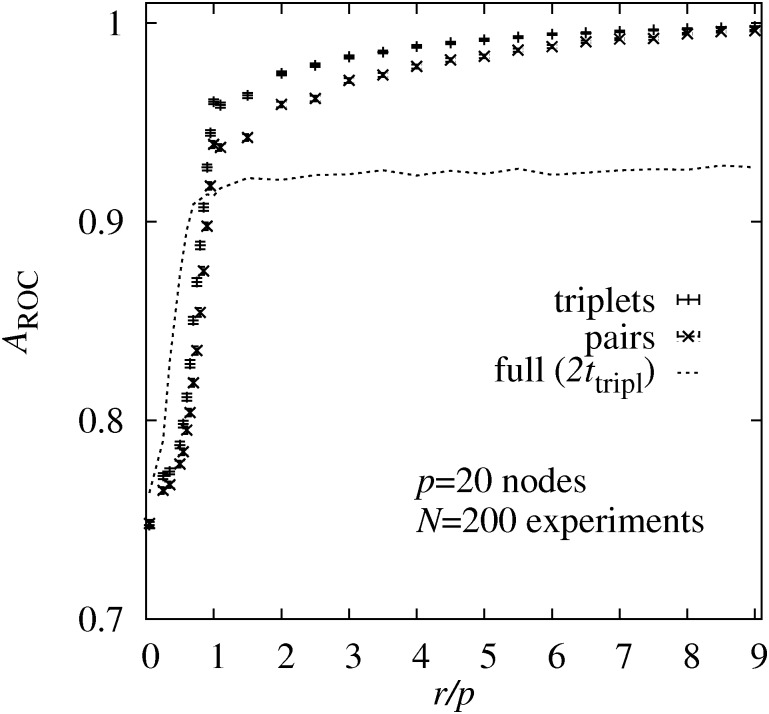
AUROC. Area *A*_ROC_ under ROC curve (AUROC) for estimating non-zero edge weights as a function of the number of interventions *r* per node. The results are obtained using three different sampling approaches using the true maximum likelihoods (**full**), the pair BS probabilities (**pair**), and the triplet BS probabilities (**triplet**). The running time for the sampling using the true maximum likelihoods was restricted to two times the CPU time of the triplet sampling.

In Fig 4 the AUROC is shown for the same data of the *p* = 20 complete DAGs. Clearly, with increasing numbers *r* of interventions, the AUROC grows. The increase is strongest for values *r* < *p*, beyond this point the increase in the quality of the estimates is much smaller. One can also observe that again the triplet-based sampling outperforms the pair-based sampling. Also, the sampling using the true maximum likelihoods, restricted to about two times the numerical effort of the triplet-based approach, is better for about *r* < 0.8*p* but worse for *r* > 0.8*p*, confirming the previous results.

**Fig 5:**
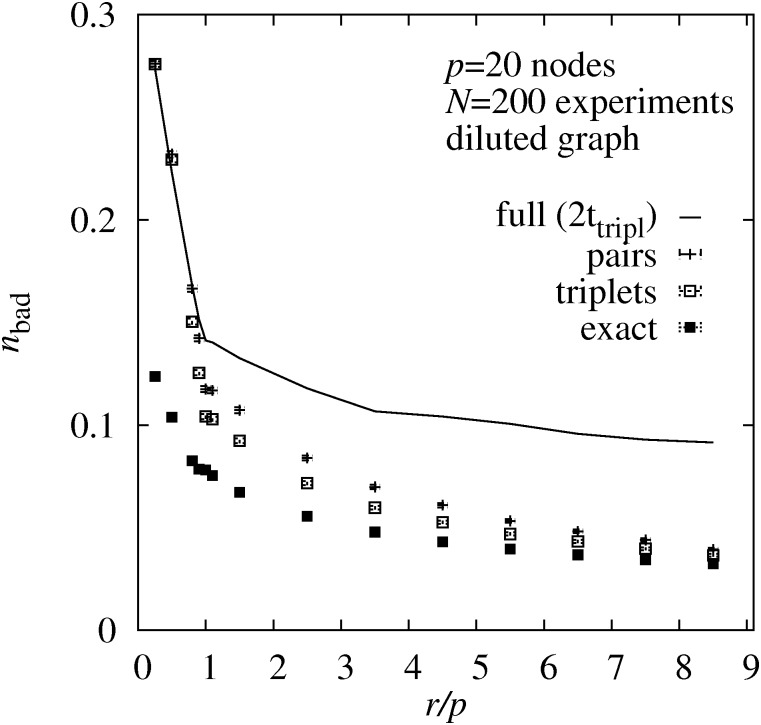
Topological errors for diluted DAGs. For a diluted graph with *p* = 20 nodes: Average fraction *n*_bad_ of incorrectly estimated edge weights as a function of the number of interventions *r* per node. The results are obtained using four different sampling approaches using the true maximum likelihoods (**full**), the pair BS probabilities (**pair**), the triplet BS probabilities (**triplet**) and using just the exact ordering of nodes of the DAGs. The running time for the sampling using the true maximum likelihoods was restricted to two times the CPU time of the triplet sampling.

We also considered diluted DAGs. In Fig 5, the number *n*_bad_ of strongly incorrectly estimated edge weights is shown as a function of relative number *r*/*p* of interventions for the case of diluted DAGs which exhibit one average *c* = 6 neighbor, which is less than one third compared to the case of the complete graphs. Here, the results of the pair and triplet-based sampling approaches are much closer to each other, but the general trend remains, showing that the triplet-based sampling outperforms the pair-based sampling, and the full likelihood-based sampling for a comparable numerical effort.

The facts that the results of the pair and triplet-based approaches are closer to each other can be some expected, because the effective number of parameters to be estimated is smaller, thus the corresponding likelihoods or probabilities will be closer to each other. Thus, we also studied larger DAGs with *p* = 50 nodes. Here we generated *N* = 500 experimental outcomes per node for each DAG. For the MCMC sampling we used again 100 independent runs for the pair-based and the triplet-based sampling, 10 independent runs for the sampling based on the true maximum likelihoods *ℓ*_max_. For the former two, we used 15100 MC steps for equilibration and 100 steps for measurement, for each of the independent runs. For the sampling based on *ℓ*_max_, due to its expensive *O*(*p*^6^) computation, we could perform only 25 MCMC steps in order to consume about two times the CPU time needed for the triplet-based sampling.

**Fig 6:**
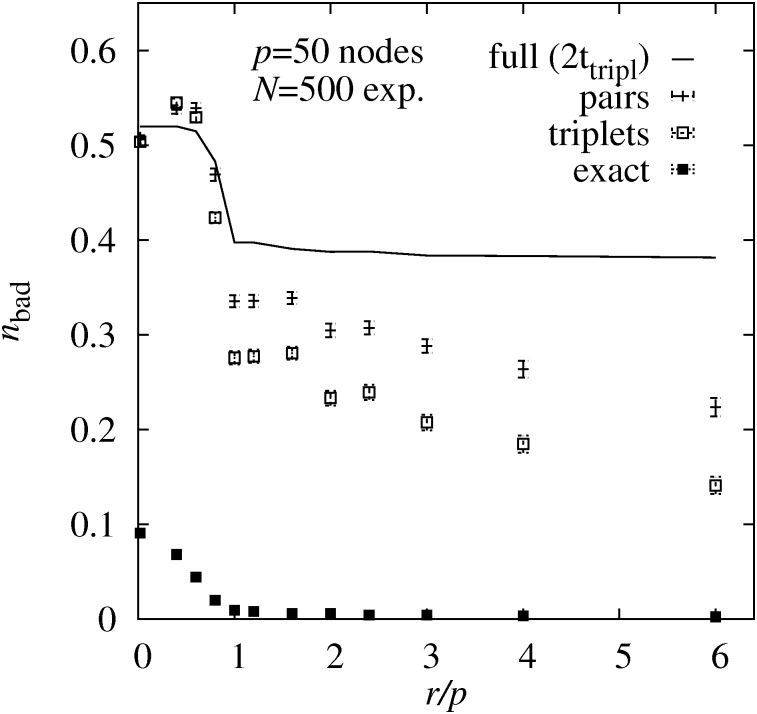
Topological errors for larger complete DAGs. Average fraction *n*_bad_ of incorrectly estimated edge weights as a function of the number of interventions *r* per node for complete graph of *p* = 50 nodes. The results are obtained using four different sampling approaches using the true maximum likelihoods (**full**), the pair BS probabilities (**pair**), the triplet BS probabilities (**triplet**) and using just the exact ordering of nodes of the DAGs. The running time for the sampling using the true maximum likelihoods was restricted to two times the CPU time of the triplet sampling.

The corresponding results of *n*_bad_(*r*/*p*) for complete graphs are shown in Fig 6. Here the differences between the approaches are indeed larger compared to the *p* = 20 case, but the general trend is confirmed that the triplet-based approach outperforms the pair-based approach, which in turn outperforms the exact sampling. The results when just using the original causal ordering form again a lower bound on what can be achieved for *n*_bad_.

### Greedy Approach

Finally, to allow for a comparison of the approaches from a different perspective, we consider the case where we do not aim at estimating parameters of the model, e.g., the weights of the causal interactions. Instead we focus on the estimation of the causal ordering itself which was used to numerically generate the data. This is a much harder task. One approach could be to enumerate all orderings and take that one exhibiting the largest maximum likelihood *ℓ*_max_ as an estimate of the correct ordering. This represents a double-nested optimization: For each given ordering, the exact maximum likelihood is obtained in a straightforward way as explained in Section “Estimating Model Parameters”. This has to be repeated for all possible orderings. Thus it would require an numerical effort *O*(*p*!) for system consisting of *p* nodes, i.e. more than exponentially.

This is not feasible for systems beyond exhibiting few nodes. Therefore, we follow a different approach here. We apply a *greedy* construction of an estimate for the true ordering.

For this purpose, we again use the pairwise and the triplet-wise probabilities, respectively. This works as follows: We initialize the ordering with a single pair (*i*, *j*) of nodes, for the pair-based approach, or the triplet (*i*, *j*, *k*) of nodes, for the triplet-based approach, which exhibits the largest value of pair preference *π*_*i*,*j*_ or the largest triplet preference *ρ*_*i*,*j*,*k*_, respectively. Next, iteratively nodes are included in the ordering, one-by-one, such that the resulting combined BS probability, evaluated according to Eqs (12) or (15), respectively, is largest. The construction is finished when a full ordering of length *p* is obtained. This means in each step, one chooses among *O*(*p*) nodes and *O*(*p*) insertion positions, i.e., one considers *O*(*p*^2^) choices. Also, like in the MCMC steps, one has to consider *O*(*p*) terms when evaluation the influence of on the pairwise likelihood for each extension of the ordering. Similarly, for the triplet-based greed approach, each insertion choice requires the calculation of *O*(*p*^2^) factors. This leads to an overall running time of *O*(*p*^3^) for the pair-based and *O*(*p*^4^) for the triplet-based greedy approaches.

To evaluate the resulting ordering, we compared it to the original ordering which was used to generate the data, while again varying the number of interventions in the same way as before. For the comparison, we used *Kendal’s tau-distance* *K*, which is defined for two orderings **o**, **o**′ as the number of pairs of nodes which appear in different relative orders in the two orderings.

$$K\left(o,o^{\prime }\right)=\left|\left\{\left\{i,j\right\}|o_{i}<o_{j}\wedge o_{i}^{\prime }>o_{j}^{\prime }\right\}\right|$$ (20)

Note that Kendal’s *tau* distance is also called *bubble-sort distance* because it states the number of elementary sorting swaps to arrange one ordering in the order of the other given ordering. The maximum possible value is *p*(*p* − 1)/2 for *p* elements.

**Fig 7:**
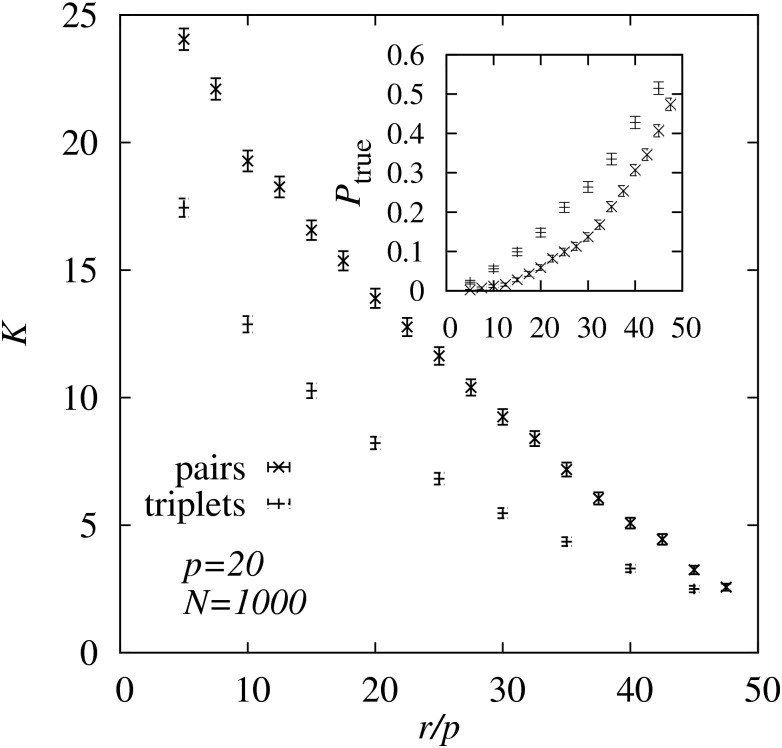
Kendall’s tau. Average Kendall’s *τ* distance *K* to the original ordering for ordering obtained via applying the greedy approach for pairs and triplet Babington-Smith probabilities. The average is obtained over 1000 DAGs of size *p* = 20, while varying the number *r* of interventions performed within the numerically generated measurement data. The inset shows the frequency *P*_true_ that the original DAG ordering is found, i.e., the frequency that *K* = 0.

In Fig 7 the average of *K* is shown for complete DAGs with *p* = 20 nodes as a function of the number *r* of interventions. Here a larger (quite unrealistic) number of *N* = 1000 experiments is numerically performed. This allowed us to change the number *r* of interventions in a very large range such that we could also access the region where the greedy approach actually determines the true ordering with high probability. One observes that indeed when increasing the number of interventions, the greedy orderings resemble the original DAG ordering more and more. Compared to the maximum value *p*(*p* − 1)/2 = 190, the orderings found by the greed approaches are quite similar to the true ordering. Interestingly, as seen in the inset of Fig 7, for about *O*(50) interventions, the greedy approaches find the true ordering, among 20! ≈ 2 × 10^18^ ones, in more than half of all cases! This is in particular striking, because apparently the numerical effort (*O*(*p*^3^) or *O*(*p*^4^)) as well as the number of interventions (linear) appears to grow only polynomially with the number of nodes. Nevertheless, for any value of *r*, the triplet-based greedy approach clearly outperforms the pair-based approach significantly. This confirms the result found above using the MCMC sampling.

## Summary and Discussion

To summarize, we studied the estimation of causal orderings and corresponding parameters in sampled data using interventions. In particular we compared pairwise Babington-Smith sampling, which was discussed before [10] with triplet-wise sampling which we introduced in this work. All results show a much better performance for the triplet sampling approach. When limiting the numerical effort to about two times the running time of the triplet sampling, a sampling using the full maximum likelihood turned out to be much worse than both pair- and triplet-wise sampling.

These results were confirmed for various cases: for data from actual biological measurements as well as for artificial data generated in a controlled way for a DAG-based Gaussian causal model. We studied small and larger DAGs, as well as completely connected and diluted ones. The general result also stays the same independently of whether one compares the estimated weight parameters directly, uses thresholding to find correct estimates, or performs an ROC analysis of the estimated nonzero weights. Also when restricting the analysis to just the prediction of the orderings, the triplet approach turns out to be much more efficient than the pair approach.

Therefore, the triplet-based approach appears to be well-balanced: It is computationally efficient enough such that long MCMC chains can be easily generated, for systems large enough for practical applications. This would be impossible when using a sampling based on the full likelihood, except for small systems. On the other hand, in combination with the final computation of the true maximum-likelihood estimators for a comparable small subset of “best” configurations, the triplet approach allows for accurate results, much better than the pair-based approach.

In principle, one could also try a similar approach based on quadruplets of nodes. Nevertheless, in contrast to when moving from pairs to triplets, we believe that this will not result in a considerable increase of accuracy. One reason, e.g., is that for the study of the Rosetta data set, the accuracy using the triplet sampling was comparable to the exact evaluation for a *finite* subset of orderings with the highest exact likelihoods (see Fig 2). One the other hand, the numerical effort for evaluating the Metropolis criterion in each MCMC step would increase to *O*(*p*^3^) for a quadruplet-based algorithm. Thus, the triplet approach seems to be multi-criterion (accuracy, numerical demand) efficient within the hierarchy of approaches based on *n*-nodes sub graphs.

Note that the sampling approaches presented here, due to the intrinsic *O*(*p*^6^) time complexity of at least the final calculations of the best-scoring orderings, are limited if applied plainly to networks of medium size. Nevertheless, it should be stressed that for any given system of *p* nodes, a complete joint maximum likelihood is calculated. There exist other approaches for the estimation of the causal structure of actually very large networks of thousands of nodes using ad hoc heuristic algorithms [16, 17] which are based, among others, on clustering approaches and work often on a coarse-grained level. Although the approaches presented here are based on generative models, allowing for probabilistic interpretations, and allowing for detailed reconstruction of the underlying networks, they can be extended to much larger systems as well. This can be achieved for a given large set of nodes by considering many different subsystems (subgraphs) of medium size, i.e., treating them with the correct joint likelihoods. The resulting sub networks can be assembled to one large consensus network. Here, e.g., the “Iterative Sub-Network Component Analysis” approach [18] or similar approaches can be applied.

Furthermore, to identify the causal structure with *large* certainty, as the present results show, a considerable number of interventions of the order of the number of nodes, is needed. Nevertheless, the approaches presented here work with any number of interventions. If only few interventions are available, the data can be modeled easily by a larger set of high-likelihood orderings. Even better, since the approaches are based on generative models with a clear probabilistic foundation, they obtained likelihoods allow to estimate how many orderings are needed to describe the data with sufficient accuracy.

On the other hand, concerning the greedy approach, much larger systems than being considered here can be easily treated using the pair- or triplets-based approximations, due to the slowly growing *O*(*p*^2^) or *O*(*p*^3^) time complexities, respectively. Nevertheless, here a large number of interventions is necessary to obtain reliable results, since one aims only at single orderings with high likelihoods, not at sample of orderings.

On the other hand, for further applications, it might be fruitful to perform a MCMC chains which consist of mixture of triplet-wise (first part of chain) and full maximum-likelihood sampling (last part). But this is beyond of the scope of the current study.

Furthermore, it could be interesting to study more thoroughly the point *r* = *p* where most results exhibit a notable change of characteristics. It could be interesting whether this change corresponds to a kind of information-driven phase transition, similar to neural networks where the memory of a network changes if the amount of data to be learned is increased beyond a threshold. We have already started research in this direction.

Finally, it should be noted that even with a powerful algorithm for causality detection, for practical applications the task it not at all straight forward. For example, when measuring gene expression data, it will depend on the method what type of data is available. When using *micro-array* data, one will obtain mRNA levels of (many) predefined targets, thus some interesting data may not be measured. On the other hand, when using a method like *RNA-seq* one will get a complete picture of the transcriptome, but one still has to preprocess the data to remove non-relevant data or add up the results for allels, if necessary. Therefore, in general, the identification of the nodes actually to be included in the DAG is always a major task, which is relevant for the actual estimation of the causal structure.

## Supporting Information

S1 Data Data Source files. This file is a tar file zipped with gzip and contains all raw data files used to generate the figures shown in this work (and a README_DATA file) which lists and explains all data files.
(TAR.GZ)

S1 Sources Program Source files. This file is a tar file zipped with gzip and contains all C programming source files to perform the simulations. Also a standard Makefile is included. Only the GNU scientific library is needed to compile. In the Makefile it is assumed that it is installed in /opt/local/lib and /opt/local/include, please change accordingly.
(TAR.GZ)
